# Supplementary material for: Tunable photo-responsive elastic metamaterials
Source: Nat Commun. 2020 May 22;11:2576. doi: 10.1038/s41467-020-16272-y (PMC7244508; doi:10.1038/s41467-020-16272-y)
Supplement: Supplementary file 1 — Supplementary Information [file 41467_2020_16272_MOESM1_ESM.pdf]

Supplementary Information

Tunable photo-responsive elastic metamaterials

A.S. Gliozzi,[1*] M. Miniaci,[2,3] A. Chiappone,[1] A. Bergamini,[2] B. Morin,[2] and E. Descrovi[1]

[1] Department of Applied Science and Technology (DISAT), Politecnico di Torino, Corso Duca degli Abruzzi 24, Torino, IT-10129, Italy.

[2] Empa, Laboratory of Acoustics and Noise Control, Überlandstrasse 129, CH-8600 Dübendorf, Switzerland.

[3] CNRS, Univ Lille, Ecole Centrale, ISEN, Univ Valenciennes, IEMN - UMR 8520, 59046 Lille cedex, France

Corresponding Author:

Antonio Gliozzi: antonio.gliozzi@polito.it

Phone: +39 011 090 7320

Fax: +39 011 090 7399

Supplementary Note 1

Photoisomerization in azobenzene moities can lead to an efficient energy transfer toward the host matrix. In order to evaluate the temperature increase induced by laser irradiation on the LRMM, thermo-camera images have been recorded as shown in Figure S1. A laser beam (λ=405nm) is directed toward a single pillar of the structure, in the same configuration described in the article main text. It is shown that the BEDA-MR is heated until a steady temperature of T~45° is reached. Due to the low thermal conductivity of the polymer, the temperature increase and the corresponding change of Young's modulus remain locally confined to the irradiated area. It is interesting to observe that no significant temperature change is observed for an irradiated BEDA sample with no MR doping (S1-d).

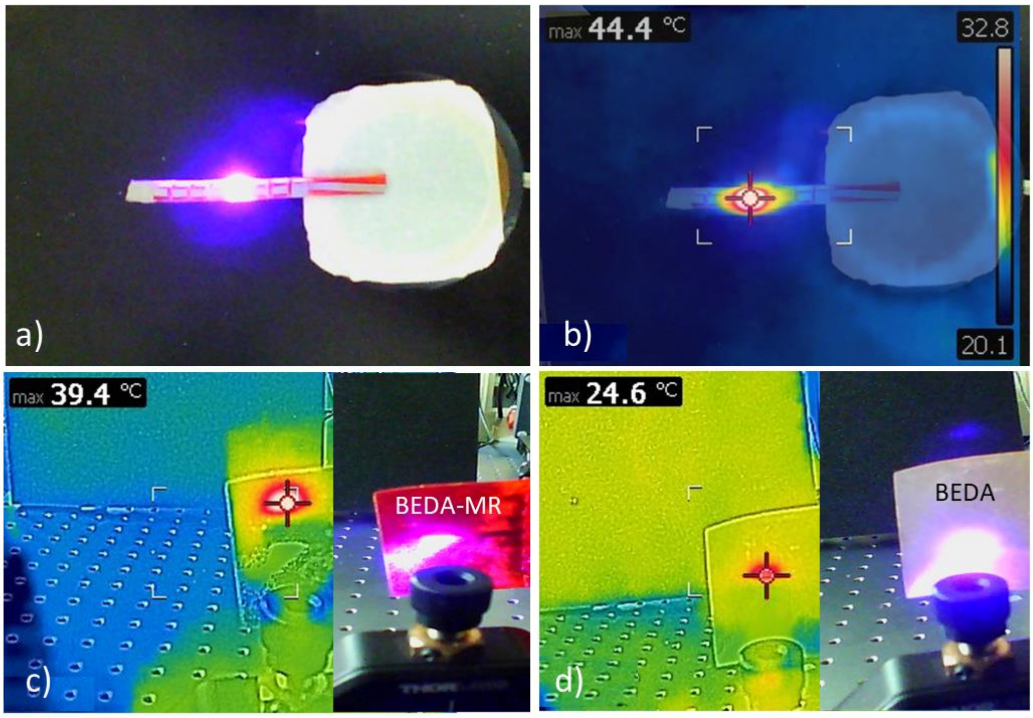

Supplementary Figure 1. (a) LRMM sample irradiated by the laser beam at a single pillar scale; (b) corresponding thermal image showing the temperature increase in the irradiated area (laser power 200 mW). (c) Photograph and thermal image of a flat BEDA-MR sample under laser irradiation; (d) Photograph and thermal image of a flat BEDA sample under laser irradiation, with no significant increase of temperature observed.

Supplementary Note 2

At temperatures below the glass transition temperature *Tg*, polymers generally exhibit an increase of Young's modulus at increasing frequencies. Furthermore, the viscoelastic properties of a polymer are generally temperature-dependent, particularly when considered close to *Tg*. A frequency and temperature-dependent characterization of a thin BEDA-MR slab have been performed.

The mechanical properties of the specimen are obtained via Dynamical Mechanical Analysis (DMA). The DMA is the state-of-art technique used for the characterization of materials with temperature/frequency dependent mechanical properties [1-3] and relies on the application of small harmonic deformations to a sample under a selection of temperatures and frequencies. This technique has allowed us to directly measure the storage and loss modulus of the material, stored as sets of isotherms. In general, the frequency range of most DMA machines, and thus the frequency span of each measured isotherm, is limited to 100 Hz. To overcome this limitation, Time-Temperature Superposition [4] has been used to estimate the material response over a larger frequency range, but at a single reference temperature.

At room temperature, the Young's modulus of the BEDA-MR sample is estimated to be about 1.78 GPa at roughly 100 kHz. At a 45° C the Young's modulus decreases to 1.25 GPa in the same frequency range, thus indicating a thermal-induced softening of the polymer.

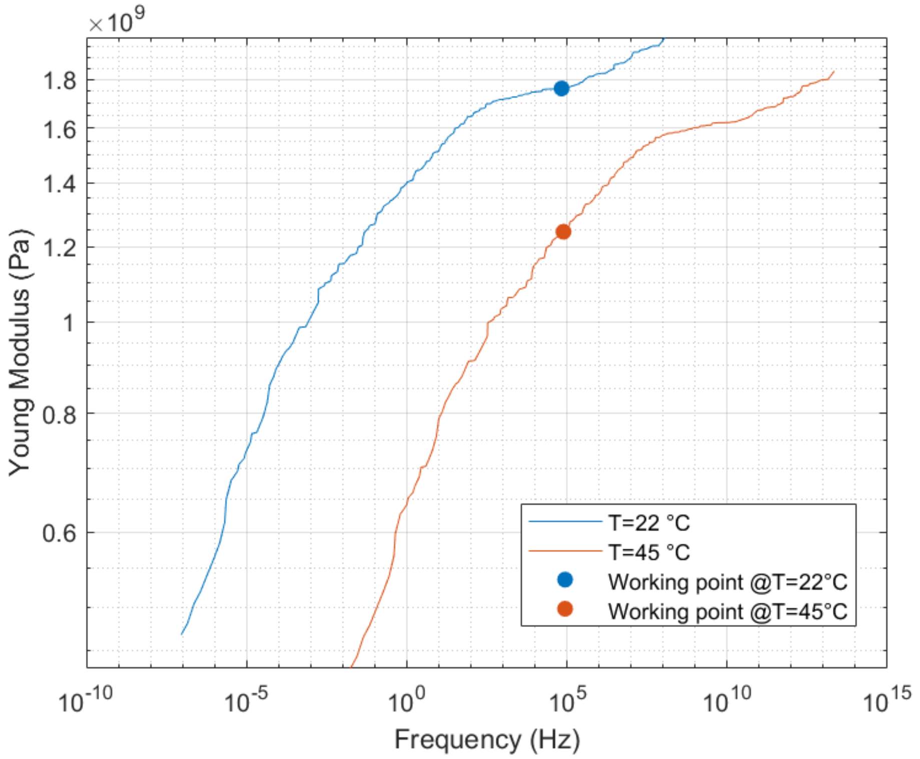

Supplementary Figure 2. Measured Young modulus of a BEDA-MR thin slab by means of Dynamical Mechanical Analysis. Values at room temperature and at 45°C are reported. Time-Temperature Superposition has been used to estimate the material response over the frequency of interest.

Supplementary Note 3

In an attempt to gain more insight on the mechanism ruling the band gap modification upon LRMM softening, we performed spectral acoustic transmission measurements in a temperature-controlled environment. In particular, we aimed at evaluating the band gap modification once the whole LRMM underwent softening, because of a general increase of temperature. As shown in Figures S3a-b, the piezo-source is contacted to the LRMM by means of an adhesive film and placed inside a temperature-controlled chamber with a transparent glass opening door. Outside the chamber, the laser interferometer is operated to perform out-of-plane vibration measurements at the free end of the LRMM, as described in the manuscript main body. Acoustic measurements are performed in a temperature range of up to T=34° C, in order to ensure a safe operation of the piezo source and the integrity of the adhesive film inside the chamber.
As shown in Figure S3c, the observed band gap is essentially rigidly shifted towards lower frequencies as the temperature increases, with a BG that shifts from 73-86 kHz at 22°C to 69-82 kHz at 34°C. This effect is quite different to the case of local illumination of individual pillars, in which a substantially wider and red-shifted band-gap is observed (as detailed in the article main body).

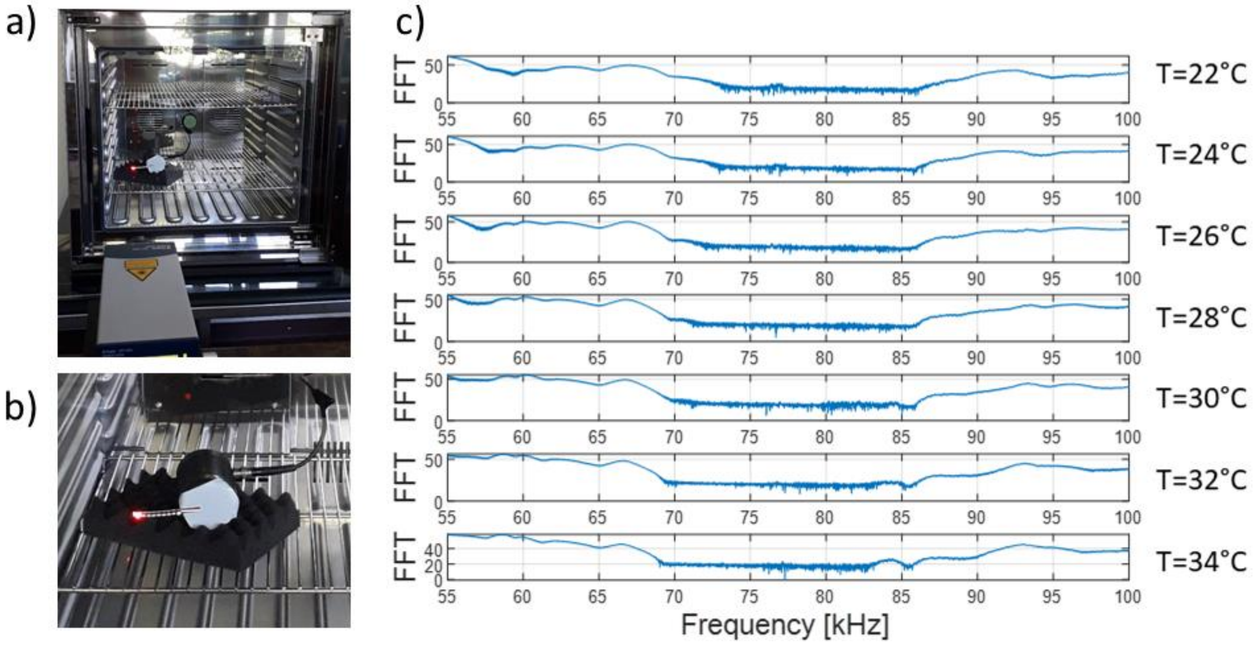

Supplementary Figure 3. (a,b) Experimental setup for the temperature-controlled acoustic transmission measurements. The piezo source and the sample are placed within a temperature-controlled and isolated environment. Laser measurements are made possible due to the presence of a transparent glass opening in the front side of the chamber. The LRMM is contacted on one end to the piezo source by means of an adhesive film. The laser interferometer is placed outside the chamber to detect the out-of-plane vibrations on the LRMM free end. c) Transmitted acoustic spectra of the LRMM at different temperatures, highlighting a shift of the BG to lower frequencies.

Supplementary Note 4

A full 3D FE model has been developed to calculate the LRMM acoustic response under different illumination conditions. The acoustic transmission spectrum in seven configurations, in which pillars from #2 to #8 are progressively softened (#2 only, #2-#3, #2-#4, etc.), is calculated. In order to take into account, the light-induced softening effect, the Young's modulus of specific pillars has been varied from 1.78 GPa to 1.25 GPa, in accordance with experimental measurements (see Supplementary Figure S2). We used a linear elastic model and kept the material damping constant in order to decouple the effect of softening and damping.
The top panel of Figure S4 shows the simulated transmission spectrum for the initial configuration, where no pillars are softened (no light). A band gap from approximately 75 kHz to 105 kHz is clearly visible. When pillar #2 is photo-softened, the low-frequency edge moves towards lower frequencies. At the same time, a strong attenuation of signals beyond 105 kHz is observed. Taking into account the additional viscoelastic damping that is realistically occurring in an experimental situation, such attenuated frequencies are likely to decrease below the noise threshold, thus giving rise to an effective widening of the band gap, as observed experimentally. These effects can be explained by taking into account the changes induced, by the softening of the modulus on the transmission spectrum of the illuminated resonator and considering the overlapping of the transmission spectrum of illuminated (red-shifted) and non-illuminated pillars. If an adjacent pillar is also softened (configuration #2-#3), this effect is further enhanced (third panel from the top of Figure S4). However, as the number of softened pillars becomes larger and comparable to the non-softened ones, the band-gap becomes reduced in width and red-shifted (configurations from #2-#4 to #2-#8), and progressively the transmitted spectrum tends to the one observed when all pillars have all undergone softening.

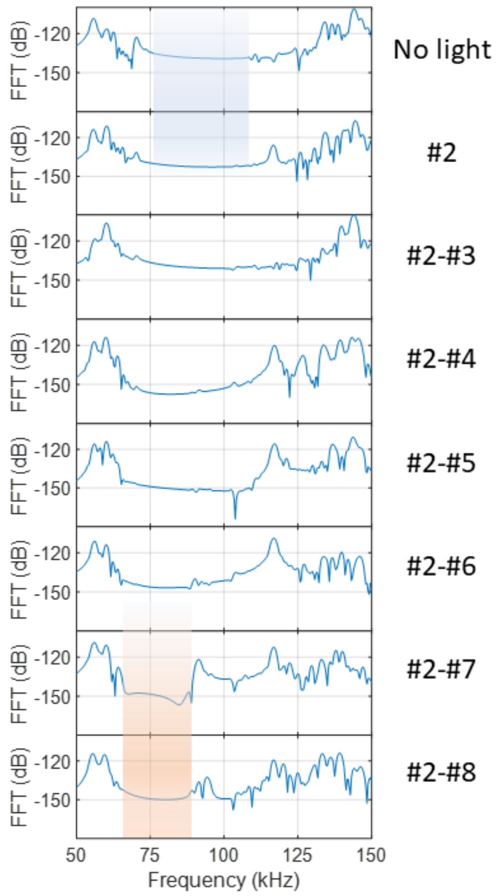

Supplementary Figure 4. LRMM acoustic transmission spectra calculated using Finite Element simulations for several configurations of softened pillars. Colored bands highlight the band gap of

the initial configuration, with no softened pillars (blue), and the final one, with pillars from #2 to #8 that have undergone softening (red).

These results point towards a mechanism mainly related to local resonance, although Bragg scattering effects cannot be ruled out. Further verification of this conclusion is under way on other configurations.

Supplementary References

[1] Menard, K.P. and Menard, N.R. (2015). Dynamic Mechanical Analysis in the Analysis of Polymers and Rubbers. In Encyclopedia of Polymer Science and Technology, (Ed.). doi:10.1002/0471440264.pst102.pub2

[2] Rouleau, Lucie, Pirk, Rogério, Pluymers, Bert, & Desmet, Wim. (2015). Characterization and Modeling of the Viscoelastic Behavior of a Self-Adhesive Rubber Using Dynamic Mechanical Analysis Tests. Journal of Aerospace Technology and Management, 7(2), 200-208. https://dx.doi.org/10.5028/jatm.v7i2.474

[3] Mano J.F., Reis R.L., Cunha A.M. (2002) Dynamic Mechanical Analysis in Polymers for Medical Applications. In: Reis R.L., Cohn D. (eds) Polymer Based Systems on Tissue Engineering, Replacement and Regeneration. NATO Science Series (Series II: Mathematics, Physics and Chemistry), vol 86. Springer, Dordrecht.

[4] Mechanical vibration and shock - Characterization of the dynamic mechanical properties of visco-elastic materials – Time-Temperature superposition. International Standard, ISO 18437-6, 2017
